# *In vitro* efficacy of four potential antimicrobial substances against *Klebsiella* species and *Streptococcus uberis* isolated from bovine mastitis

**DOI:** 10.3389/fvets.2026.1782821

**Published:** 2026-03-17

**Authors:** Jennifer Hillebrand, Hilke Oltmanns, Jessica Meißner

**Affiliations:** Department of Pharmacology, Toxicology and Pharmacy, University of Veterinary Medicine Hannover, Hanover, Germany

**Keywords:** bovine mastitis, *Klebsiella* species, methylglyoxal, N-acetylcysteine, N-chlorotaurine, polyvinylpyrrolidone iodine, *Streptococcus uberis*

## Abstract

Mastitis is an important disease in dairy cattle with huge influence on animal welfare and economic situation of farmers. With very limited choices in antibiotic therapy of bovine mastitis and the relevance of emerging antimicrobial resistances, discovery of new antimicrobials is one of the major challenges for veterinary pharmacological research. Thus, *in vitro* efficacy of four promising substances was investigated: polyvinylpyrrolidone iodine (PVP iodine), N-acetylcysteine (NAC), N-chlorotaurine (NCT), and methylglyoxal (MGO). PVP iodine is an antiseptic commonly used in all medical fields. The two amino acid derivates NAC and NCT are a potent antioxidant respective oxidant with antimicrobial properties in various indications. MGO is one of the active components in Manuka honey, causing bacterial death by repression of protein and nuclei acid synthesis. For experiments, *Klebsiella* species (spp.) and *Streptococcus (S.) uberis* were isolated from bovine milk samples. Subsequently, broth microdilutions with common antibiotic agents and the four potential antimicrobials for determination of minimal inhibitory concentrations (MICs) and examinations regarding minimal bactericidal concentrations (MBCs), also time-dependent, were performed according to CLSI standards. Regarding antimicrobial susceptibility, growth of *Klebsiella* spp. isolates was not restrained by examined concentrations of benzylpenicillins, aminopenicillins, isoxazolyl penicillins, macrolides, and lincosamides. However, studied concentrations of aminopenicillins combined with beta-lactamase inhibitors, cephalosporins (partly combined with aminoglycosides) as well as fluoroquinolones inhibited their growth. In contrast, all *S. uberis* isolates were susceptible to tested concentrations of penicillins, cephalosporins, macrolides, lincosamides, and fluoroquinolones. Broth microdilutions of the four substances revealed median MICs of PVP iodine with 5 mg/mL for *Klebsiella* spp. and 4 mg/mL for *S. uberis* and NAC with MICs of 5 mg/mL for *Klebsiella* spp. and 1.75 mg/mL for *S. uberis.* MICs of NCT were 1.5 mg/mL for *Klebsiella* spp. and 1 mg/mL for *S. uberis.* The lowest MICs were found for MGO with 0.4 mg/mL for *Klebsiella* spp. and 0.1375 mg/mL for *S. uberis*. Growth of every bacterial isolate was inhibited through all four substances, even when resistant to commonly used antibiotic agents. Therefore, with positive findings in further experiments regarding biocompatibility and efficacy, they could be considered as auspicious alternatives for mastitis treatment in dairy cattle.

## 1 Introduction

Bovine mastitis remains one of the most significant diseases affecting the global dairy industry (1), posing substantial threats to both animal welfare and economic stability. According to a large German prevalence study, almost 20% of dairy cows showed clinical mastitis without and even 5% clinical mastitis with impaired general condition per year (2). The financial burden of the disease is multifaceted, comprising direct costs from reduced milk production, discarded milk, and veterinary treatments, as well as indirect, long-term costs such as decreased herd-level fertility and increased culling rates (3). On average, a single case of clinical mastitis can incur costs of approximately €224, with infections caused by gram-negative bacteria being particularly costly at an estimated €457 per case (4).

Mastitis is caused by a wide array of over 130 different microorganisms (5). However, the primary etiological agents are broadly categorized as gram-positive and gram-negative bacteria. Key gram-positive pathogens include *Staphylococcus* species and environmental streptococci, notably *Streptococcus uberis* (*S. uberis*). Among gram-negative bacteria, coliforms such as *Escherichia coli* (*E. coli*) and *Klebsiella* species (*Klebsiella* spp.) are major contributors (6).

This study focuses on two particularly relevant pathogens: *S. uberis* and *Klebsiella* spp. *S. uberis* is a predominant environmental pathogen, accounting for a significant portion of mastitis cases, with a prevalence of 20.2% reported in northern Germany (7). Meanwhile, *Klebsiella* spp. are emerging as pathogens of increasing concern. Surveillance data from southern Germany (2014–2022) indicates a rising number of coliform isolates, with *Klebsiella* spp. [including *Klebsiella* (*K.*) *pneumoniae* and *K. oxytoca*] constituting 9% of these isolates (8). Mastitis induced by *Klebsiella* spp. is often more severe and prolonged compared to that caused by *E. coli*, leading to more significant reductions in milk production and higher culling risks (9–12).

*S. uberis* causes mild to moderate (88.2%) as well as severe (11.8%) mastitis. As a result of its widespread distribution, 26.5% of severe mastitis cases in northern Germany were caused by *S. uberis*, whereas 30.5% were due to coliforms (7). *S. uberis* induced mastitis emerged significantly more frequently in the first than in later lactations (7). Ability of internalization of *S. uberis* into mammary epithelial cells and formation of abscesses leads to chronic and recurrent intramammary infections and impedes success of antibiotic therapy (13).

Antibiotic therapy of bovine mastitis is still the most frequent choice (14). In 2024, 36.5 t antibiotics were used for treatment of dairy cows in Germany (15). Selective therapy of clinical mastitis achieves more relevance, as not every intramammary infection benefits from antibiotic treatment in terms of improved cure rates. Selection of antimicrobial agent and application route (parenterally or intramammary) underlies several criteria, such as severity of mastitis and detected bacteria (16).

While antibiotic treatment of mild or moderate intramammary infections with *E. coli* is not recommended, due to high spontaneous cure rates (7, 17), antibiotic treatment of *Klebsiella* spp. induced mastitis achieved higher bacterial cure than untreated controls (9, 18).

In contrast, antimicrobial therapy of mastitis caused by gram-positive bacteria as *Streptococcus* species is particularly indicated, as it improves bacteriological cure significantly (19).

Regarding antimicrobial resistance and development of minimal inhibitory concentration (MIC) values, in northern Germany rising MICs for gram-negative and some gram-positive mastitis pathogens like *S. uberis* were reported (20). A national antimicrobial resistance surveillance program for animal pathogens found steadily increasing resistance from *S. uberis* to tetracycline from around 20% (2002) to 45% (2021) (21). Similarly, resistance from mastitis associated *E. coli* to trimethoprim/sulfamethoxazole almost doubled within 4 years from below 10% (2018) to 19% (2022). Ampicillin and doxycycline resistance from *E. coli* followed the upward trend over the same time (22).

The conventional treatment for bovine mastitis relies heavily on antibiotic therapy. However, the limited availability of effective antibiotics and the global rise of antimicrobial resistance (AMR) present major challenges for veterinary medicine. This situation necessitates the exploration of novel, non-antibiotic antimicrobial agents that can effectively control mastitis pathogens, including those that are resistant to conventional drugs.

In response to this need, this study investigates the *in vitro* efficacy of four promising non-antibiotic substances against isolates of *Klebsiella* spp. and *S. uberis* from bovine mastitis cases. The selected candidates are: polyvinylpyrrolidone iodine (PVP iodine), N-acetylcysteine (NAC), N-chlorotaurine (NCT) and methylglyoxal (MGO).

PVP iodine is a commonly used antiseptic in all medical fields. Due to complex formation with molecular iodine (I_2_), membrane transport is simplified and antimicrobial activity increased. Cytotoxicity seems to depend on concentration and tissue type (23).

The second substance, NAC, is a derivate of amino acid L-cysteine. As mucolytic agent and antidot to acetaminophen intoxication, it is approved by the Food and Drug Administration. Its antimicrobial properties result from reduction of disulfide bonds, oxidant scavenging and biofilm destruction (24).

Another amino acid derivate, NCT, is produced endogenously by activated granulocytes and monocytes through halogenation of taurine. The synthetic sodium salt enables storage and targeted therapeutic use. Efficacy against various bacteria, fungi, viruses, and parasites has been proved. Clinical studies showed high tissue tolerability (25).

The last substance is of natural origin: MGO is one of the active components in Manuka honey, originating from nectar of *Leptospermum scoparium*. It causes bacterial death by membrane depolarization and repression of protein and nuclei acid synthesis (26).

The primary objective was to determine the Minimal Inhibitory Concentrations (MICs) of these four substances against clinically relevant mastitis pathogens. By evaluating their potential, this research aims to identify auspicious alternatives for the future treatment of bovine mastitis, thereby addressing the critical need for new therapeutic options in an era of growing antimicrobial resistance.

## 2 Materials and methods

### 2.1 Collection and identification of bacterial strains

*Klebsiella* spp. strains were isolated from quarter milk samples of dairy cows with clinical mastitis collected within routine diagnostics prior to treatment from two livestock veterinarians in Northern Germany. Each sample originated from a different farm, cow and quarter. Milk samples were cultured on selective agar plates VetoRapid (Vetoquinol, Ismaning, Germany) at 37 °C in ambient air for 24 h. After incubation, colonies were morphologically pre-selected: according to the manufacturer’s instructions, other coliforms like *Klebsiella* spp. appear as red or violet colonies in sector one of the agar plates. In Amies gel with charcoal swabs (Heinz Herenz, Hamburg, Germany), bacteria were transported to the laboratory and streaked on Columbia sheep blood (COL) agar plates (Thermo Fisher Scientific, Darmstadt, Germany). Identification was performed with matrix assisted laser desorption ionization time of flight mass spectrometry (MALDI-TOF MS; Bruker, Bremen, Germany) by the Institute of Microbiology, University of Veterinary Medicine Hannover, using the BDAL V13 database version 7 and score thresholds above 2.3 for species-level identification.

*S. uberis* isolates were collected from quarter milk samples of udders from lactating Holstein-Friesian cows. Udders were obtained from a slaughterhouse in North Rhine-Westphalia, Germany, for experiments with mammary tissue. Further information about the origin of the udders except for the breed of the cow were not available. Prior to experiments, milk was examined visually for abnormalities to identify infected quarters. Quarters with anomalous secretion were selected for sample collection and teats were cleaned with damp wipes and disinfected with 70% ethanol. COL agar plates were inoculated with the milk and cultured at 37 °C in ambient air for 24 h. For achievement of a pure culture to ensure accurate identification, subcultures of single colonies, picked by colony morphology, were streaked on COL agar and incubated for another 24 h until identification, which was performed as mentioned above for *Klebsiella* spp.

Three *K. oxytoca,* seven *K. pneumoniae* and eight *S. uberis* strains were isolated and cryopreserved in tryptic soy broth (Oxoid, Hampshire, England) supplemented with glycerol at –70 °C. For the following experiments, three *K. oxytoca,* six *K. pneumoniae* and six *S. uberis* isolates were randomly selected out of this collection.

Type strains *K. pneumoniae ssp. pneumoniae* DSM 30104 and *S. uberis* DSM 20569 (DSMZ – German collection of microorganisms and cell cultures GmbH, Braunschweig, Germany) were used as control strains for subsequent examinations.

### 2.2 Antimicrobial susceptibility testing

Antimicrobial susceptibility was examined with commercially available 96 well microtiter plates MICRONAUT-S Mastitis (Bruker, Bremen, Germany) according to the manufacturer’s instructions and CLSI standard M07-A9 (27). The minimal inhibitory concentrations (MICs) of 14 antibiotic agents from the following groups were determined: penicillins (penicillin G, 0.125 to 8 μg/mL; ampicillin, 0.25 to 32 μg/mL; oxacillin, 0.25 to 2 μg/mL), cephalosporins of the first (cefazolin, 2 to 8 μg/mL), second (cefoxitin, 4 μg/mL), third (cefoperazone, 0.5 to 8 μg/mL; ceftiofur, 2 to 4 μg/mL) and fourth (cefquinome, 2 to 8 μg/mL) generation, macrolides (erythromycin, 0.25 to 4 μg/mL), lincosamides (pirlimycin, 2 to 4 μg/mL), fluoroquinolones (marbofloxacin, 1 to 4 μg/mL) and combinations (amoxicillin/clavulanic acid, 8/4 to 32/16 μg/mL; penicillin/novobiocin, 1/2 to 4/8 μg/mL; kanamycin/cefalexin, 2/0.2 to 16/1.6 μg/mL) (Supplementary Table 1). The MIC is defined as the lowest concentration of an antimicrobial agent which is able to inhibit bacterial growth (27).

Prior to experiments, bacteria were streaked onto COL agar plates and incubated for 18 to 24 h. The next day, isolated colonies were transferred into 0.9% NaCl solution to achieve a turbidity equivalent to McFarland (McF) Standard of 0.5. In a second step, the inoculum was diluted 1:200 for *Klebsiella* spp. and 1:50 for *S. uberis* in 0.9% NaCl solution. 90 μL cation adjusted Mueller-Hinton broth (CAMBH; Roth, Karlsruhe, Germany) and 10 μL diluted bacterial suspension were delivered into every well. After 18 to 20 h of incubation at 37 °C in ambient air, results were read visually. The MIC is defined as the lowest concentration without visible bacterial growth; visible bacterial growth is defined as definite turbidity or button identical in occurrence with the positive control (27). Antimicrobial susceptibility testing with three *K. oxytoca,* six *K. pneumoniae*, six *S. uberis* isolates and the two type strains was processed once, as usual in routine diagnostics.

### 2.3 Minimal inhibitory concentrations of potential antimicrobials

Subsequently, broth microdilutions of *Klebsiella* spp. and *S. uberis* were performed according to CLSI standard M07-A9 (27) to determine MICs of PVP iodine (Sigma-Aldrich, Steinheim, Germany), NAC (Sigma-Aldrich, Steinheim, Germany), NCT (Alchemist, Stuttgart, Germany) and MGO (Thermo Fisher Scientific, Darmstadt, Germany). Similar to the antimicrobial susceptibility testing, a suspension with a turbidity equivalent to McF standard 0.5 in NaCl was prepared. 10 μL of the inoculum and 90 μL of the selected substances diluted in CAMBH were transferred into 96 well round bottom microtiter plates (Greiner, Frickenhausen, Germany) and incubated for 16 to 20 h at 37 °C in ambient air. As positive control, 10 μL bacterial suspension and 90 μL CAMBH were used, as negative control 100 μL CAMBH and 100 μL of the substance solution of each concentration were used. For PVP iodine, 1 to 10 mg/mL, NAC 0.5 to 5 mg/mL, NCT 0.5 to 10 mg/mL and MGO 0.05 to 0.8 mg/mL were investigated (Supplementary Table 2). Results were read visually. The MIC is defined as the lowest concentration without visible bacterial growth, visible bacterial growth is defined as definite turbidity or button identical in occurrence with the positive control (27).

Broth microdilutions were conducted with three *K. oxytoca,* six *K. pneumoniae*, six *S. uberis* isolates and the two type strains. Each combination of substance and isolate or type strain was repeated six times (*n* = 6).

### 2.4 Minimal bactericidal concentrations of potential antimicrobials

Determination of minimal bactericidal concentrations (MBCs) of NAC, NCT, and MGO for *Klebsiella* spp. and *S. uberis* was conducted in accordance with CLSI standard M26-A (28).

Stock solutions of the antimicrobials in sterile phosphate-buffered saline (PBS; pH 7.4) were prepared and diluted in PBS to the required concentrations; 250 μL of the solution was added to 250 μL of a bacterial suspension in PBS with a turbidity equivalent to McF standard 0.5 and incubated in ambient air at room temperature for 10 min. Following, 100 μL of this inoculum were added to 900 μL of Dey–Engley neutralizing broth (Merck KGaA, Darmstadt, Germany) for NAC and MGO and incubated for another 5 min at room temperature. For NCT, an adapted neutralizing broth (29), consisting of 1% L-methionine (Sigma-Aldrich, Steinheim, Germany) and 1% L-histidine (Sigma-Aldrich, Steinheim, Germany) in bidestilled water with 1% hydrochloric acid for solubility, was used. Afterwards, tenfold dilutions of the inoculum in PBS were prepared and 100 μL of each dilution level were plated onto COL agar plates in duplicate. As positive controls (one for each neutralizing broth), the procedure as described above was carried out with 250 μL PBS instead of the antimicrobial agent. The negative controls were 100 μL stock solution of each substance or neutralizing broth. After incubation of 24 h for *Klebsiella* spp. and 48 h for *S. uberis* in ambient air at 37 °C, colony forming units (CFU) were counted and reduction of bacterial concentration in % in relation to the positive control was calculated. The MBC is defined as a reduction of bacterial concentration over three logarithmic steps (99.9%).

Concentration ranges of 0.5 to 10 mg/mL for NAC, 1 to 45 mg/mL for NCT and 1 to 50 mg/mL for MGO and were examined.

MBCs were performed with one *K. oxytoca,* one *K. pneumoniae* and one *S. uberis* isolate (all randomly selected) once as preliminary examination for assessment of concentrations for the time kill assay.

### 2.5 Time-kill assay of potential antimicrobials

A time-kill assay for the potential antimicrobials was performed as described above for the MBCs with little variation: instead of 10 min of incubation, bacteria and antimicrobials were incubated for 2, 5, or 10 min before neutralization. Similarly, the reduction of the bacterial concentration in % compared to the positive control of each time point was calculated.

Examined concentrations were based on findings from the MBC assay: for NAC, 3.75 to 6.5 mg/mL, NCT 40 to 47 mg/mL and MGO 23 to 30 mg/mL were tested. Time points of 2 and 5 min were examined for all concentrations, 10 min only if the concentration was not tested in the MBC assay before.

Time-kill assays were conducted once for the same *K. oxytoca, K. pneumoniae* and *S. uberis* isolate used for the MBCs.

## 3 Results

### 3.1 Antimicrobial susceptibility testing

#### 3.1.1 *Klebsiella* species

The median MICs of 14 antibiotic agents for *K. oxytoca* and *K. pneumoniae* isolates compared to type strain *K. pneumoniae ssp. pneumoniae* DSM 30104 are shown in Table 1.

**Table 1:** Median minimal inhibitory concentrations (MICs) of 14 antibiotic agents for three *Klebsiella oxytoca* and six *Klebsiella pneumoniae* isolates compared to type strain *Klebsiella pneumoniae ssp. pneumoniae* DSM 30104.

Antibiotic agent	Median MICs (μg/mL)		
	*K. oxytoca*	*K. pneumoniae*	*K. pneumoniae* DSM 30104
Penicillin G	>8	>8	>8
Penicillin/novobiocin	>4/8	>4/8	>4/8
Ampicillin	32 (>32)	32	32
Amoxicillin/clavulanic acid	≤8/4	≤8/4	≤8/4
Oxacillin	>2	>2	>2
Kanamycin/cefalexin	≤2/0.2 (>16/1.6)	≤2/0.2	≤2/0.2
Cefazolin	4 (>8)	≤2 (4)	4
Cefoxitin	≤4	≤4 (>4)	>4
Cefoperazone	1 (>8)	≤0.5	≤0.5
Ceftiofur	≤2 (>4)	≤2	≤2
Cefquinome	≤2 (8)	≤2	≤2
Erythromycin	>4	>4	>4
Pirlimycin	>4	>4	>4
Marbofloxacin	≤1	≤1	≤1

Median minimal inhibitory concentrations in μg/mL, numbers in parentheses show deviations from median of single isolates.

Distribution of MICs of antibiotic agents was within a small range, as almost all MICs for *K. oxytoca* and *K. pneumoniae* isolates and the type strain were identical. Individual MICs of isolates and type strains can be found in Supplementary Table 3.

For the following antibiotic agents, MICs for *Klebsiella* spp. were higher than the concentrations examined: penicillin G (>8 μg/mL), penicillin/novobiocin (>4/8 μg/mL), oxacillin (>2 μg/mL), erythromycin (>4 μg/mL), and pirlimycin (>4 μg/mL). In contrast, subsequent MICs for *Klebsiella* spp. were lower than concentrations tested of the antibiotic agents: amoxicillin/clavulanic acid (≤8/4 μg/mL), kanamycin/cefalexin (≤2/0.2 μg/mL), cefoxitin (≤4 μg/mL), ceftiofur (≤2 μg/mL), cefquinome (≤2 μg/mL), and marbofloxacin (≤1 μg/mL). For ampicillin, median MICs for *Klebsiella* spp. were 32 μg/mL. Differences in MICs between *K. oxytoca* and *K. pneumoniae* isolates and the type strain were only found for cephalosporines of first to third generation. Median MICs of cefazolin, a first-generation cephalosporin, differed between *K. pneumoniae* (≤2 μg/mL) and *K. oxytoca* (4 μg/mL). Similarly, median MICs of cefoperazone, belonging to the third generation of cephalosporins, were lower for *K. pneumoniae* with ≤0.5 μg/mL than for *K. oxytoca* with 1 μg/mL. The only deviation of MICs between isolates and type strain can be found for cefoxitin, a second-generation cephalosporin, where 4 μg/mL inhibit growth of *K. oxytoca* and *K. pneumoniae* isolates, but not of the type strain.

One *K. oxytoca* isolate was only susceptible to 4 of 14 examined antibiotic agents: inhibition of growth was only reached through amoxicillin/clavulanic acid, cefoxitin, cefquinome, and marbofloxacin (Table 1; Supplementary Table 3).

Taken together, *Klebsiella* spp. isolates were not susceptible to examined concentrations of benzylpenicillins, aminopenicillins, isoxazolyl penicillins, macrolides, and lincosamides.

However, studied concentrations of aminopenicillins combined with beta-lactamase inhibitors, cephalosporins of first generation (partly combined with aminoglycosides), second, third and fourth generation as well as fluoroquinolones inhibited their growth.

#### 3.1.2 Streptococcus uberis

In Table 2, the median MICs of 14 antibiotic agents for *S. uberis* isolates and type strain *S. uberis* DSM 20569 are listed.

**Table 2:** Median minimal inhibitory concentrations (MICs) of 14 antibiotic agents for six *Streptococcus uberis* isolates compared to type strain *Streptococcus uberis* DSM 20569.

Antibiotic agent	Median MICs (μg/mL)	
	*S. uberis*	*S. uberis* DSM 20569
Penicillin G	≤0.125	≤0.125
Penicillin/novobiocin	≤1/2	≤1/2
Ampicillin	≤0.25	≤0.25
Amoxicillin/clavulanic acid	≤8/4	≤8/4
Oxacillin	≤0.25	≤0.25
Kanamycin/cefalexin	≤2/0.2	≤2/0.2
Cefazolin	≤2	≤2
Cefoxitin	≤4	≤4
Cefoperazone	≤0.5 (1)	≤0.5
Ceftiofur	≤2	≤2
Cefquinome	≤2	≤2
Erythromycin	≤0.25	≤0.25
Pirlimycin	≤2 (4)	≤2
Marbofloxacin	≤1	≤1

Median minimal inhibitory concentrations in μg/mL, numbers in parentheses show deviations from median of single isolates.

Except for cefoperazone and pirlimycin, MICs did not vary among the *S. uberis* isolates and were identical with the type strain. Individual MICs can be found in Supplementary Table 4. Median MICs for *S. uberis* were lower than the examined concentrations of all fourteen antibiotic agents. All *S. uberis* isolates and the type strain were susceptible to tested concentrations of penicillins, cephalosporins of first to fourth generation, macrolides, lincosamides, and fluoroquinolones.

### 3.2 Minimal inhibitory concentrations of potential antimicrobials

#### 3.2.1 *Klebsiella* species

Table 3 shows the median MICs of PVP iodine, NAC, NCT, and MGO for *K. oxytoca* and *K. pneumoniae* compared to the median MICs of the type strain *K. pneumoniae ssp. pneumoniae* DSM 30104. Individual MICs can be found in Supplementary Table 5.

**Table 3:** Median minimal inhibitory concentrations (MICs) of four potential antimicrobial substances for three *Klebsiella oxytoca* and six *Klebsiella pneumoniae* isolates compared to type strain *Klebsiella pneumoniae ssp. pneumoniae* DSM 30104.

Substance	Median MICs (mg/mL)		
	*K. oxytoca*	*K. pneumoniae*	*K. pneumoniae* DSM 30104
PVP iodine	6 (4–6)	5 (4–6)	4 (4–5)
NAC	4	5 (4–5)	4
NCT	1 (1–1.5)	1.5 (1–2)	1 (1–1.5)
MGO	0.35 (0.3–0.5)	0.4 (0.2–0.5)	0.4 (0.3–0.5)

Median minimal inhibitory concentrations in mg/mL, numbers in parentheses show range; *n* = 6 for every combination of substance and isolate/type strain; PVP iodine, polyvinylpyrrolidone iodine; NAC, N-acetylcysteine; NCT, N-chlorotaurine; MGO, methylglyoxal.

In broth microdilutions of PVP iodine, MICs varied from 4 to 6 mg/mL with a median MIC of 6 mg/mL for *K. oxytoca* and 5 mg/mL for *K. pneumoniae,* compared to 4 mg/mL for the type strain.

Regarding NAC, MICs were between 4 to 5 mg/mL with median MICs of 4 mg/mL for *K. oxytoca* and the type strain in contrast to 5 mg/mL for *K. pneumoniae*.

Furthermore, MICs for NCT were between 1 to 2 mg/mL with median MICs of 1 mg/mL for *K. oxytoca* and the type strain, similar to 1.5 mg/mL for *K. pneumoniae*.

MGO’s MICs ranged from 0.2 to 0.5 mg/mL with median MICs of 0.35 mg/mL for *K. oxytoca* and 0.4 mg/mL for *K. pneumoniae* and the type strain.

#### 3.2.2 Streptococcus uberis

Table 4 compares median MICs of PVP iodine, NAC, NCT, and MGO for *S. uberis* isolates and the type strain *S. uberis* DSM 20569. Individual MICs can be found in Supplementary Table 6.

**Table 4:** Median minimal inhibitory concentrations (MICs) of four potential antimicrobial substances for six *Streptococcus uberis* isolates compared to type strain *Streptococcus uberis* DSM 20569.

Substance	Median MICs (mg/mL)	
	*S. uberis*	*S. uberis* DSM 20569
PVP iodine	4 (2–5)	3 (2–3)
NAC	1.75 (1.5–2.5)	≤0.5
NCT	1 (1–3)	1
MGO	0.1375 (0.1–0.25)	0.1

Median minimal inhibitory concentrations in mg/mL, numbers in parentheses show range; *n* = 6 for every combination of substance and isolate/type strain; PVP iodine, polyvinylpyrrolidone iodine; NAC, N-acetylcysteine, NCT, N-chlorotaurine, MGO, methylglyoxal.

For PVP iodine, MICs ranged from 2 to 5 mg/mL, with median MICs of 4 mg/mL for *S. uberis* isolates and 3 mg/mL for the type strain.

Besides, *S. uberis* isolates had MICs between 1.5 to 2.5 mg/mL for NAC with a median MIC of 1.75 mg/mL, differing from ≤0.5 mg/mL for the type strain.

MICs for NCT varied from 1 to 3 mg/mL with a median MIC of 1 mg/mL for *S. uberis* isolates and type strain.

Concerning MGO, deviations from the median MIC are from 0.1 to 0.25 mg/mL with median MICs of 0.1375 mg/mL for *S. uberis* isolates and 0.1 mg/mL for the type strain.

Altogether, all isolates and type strains were susceptible to every test substance. MICs ranged within two dilution levels around the median. Comparing median MICs of the isolates and the type strains, they are identical or differ by one dilution level, except for NAC and *S. uberis*.

Between *Klebsiella* spp. and *S. uberis*, median MICs of PVP iodine and NCT for *Klebsiella* spp. are approximately one dilution level higher than for *S. uberis*, in contrast to MGO and NAC, where the median MICs differ by three respective five dilution levels.

### 3.3 Minimal bactericidal concentrations of potential antimicrobials

#### 3.3.1 *Klebsiella* species

In Table 5, MBCs of NAC, NCT, and MGO for *K. oxytoca* and *K. pneumoniae* are presented.

**Table 5:** Minimal bactericidal concentrations (MBCs) of three potential antimicrobial substances for one *Klebsiella oxytoca* and one *Klebsiella pneumoniae* isolate and reduction of bacterial concentration compared to positive control.

Substance	MBCs (mg/mL)			
	*K. oxytoca*		*K. pneumoniae*	
	Concentration	Reduction	Concentration	Reduction
NAC	6.5	99.96	6.5	99.99
NCT	45	99.68	40	99.74
MGO	28	99.66	30	98.36

Minimal bactericidal concentrations in mg/mL and reduction of bacterial concentration in % compared to positive control; *n* = 1 for every combination of substance and isolate; NAC, N-acetylcysteine; NCT, N-chlorotaurine; MGO, methylglyoxal.

6.5 mg/mL of NAC reduced bacterial concentrations of *K. oxytoca* and *K. pneumoniae* about 99.96 and 99.99%, respectively, and can therefore be considered as MBC for this substance and examined in the time-kill assay.

For NCT, 45 mg/mL for *K. oxytoca* and 40 mg/mL for *K. pneumoniae* were slightly below the required reduction of 99.9% (99.68 and 99.74%).

Similarly, MGO with 28 mg/mL for *K. oxytoca* (99.66%) and 30 mg/mL for *K. pneumoniae* (98.36%) did not show a sufficient reduction of bacterial concentrations compared to the positive control.

Therefore, concentrations of both substances were mildly elevated in the time-kill assay.

#### 3.3.2 Streptococcus uberis

Table 6 shows MBCs of NAC, NCT, and MGO for *S. uberis.*

**Table 6:** Minimal bactericidal concentrations (MBCs) of three potential antimicrobial substances for one *Streptococcus uberis* isolate and reduction of bacterial concentration compared to positive control.

Substance	MBCs (mg/mL)	
	*S. uberis*	
	Concentration	Reduction
NAC	3.75	99.94
NCT	40	99.94
MGO	25	99.99

Minimal bactericidal concentrations in mg/mL and reduction of bacterial concentration in % compared to positive control; *n* = 1 for every combination of substance and isolate; NAC, N-acetylcysteine; NCT, N-chlorotaurine; MGO, methylglyoxal.

All examined concentrations of NAC, NCT and MGO reduced bacterial growth of *S. uberis* more than 99.9% compared to the positive control. The MBCs for these substances are consequently 3.75 mg/mL for NAC, 40 mg/mL for NCT and 25 mg/mL for MGO and were examined further in the time-kill assay. Bactericidal activity of all three substances against *S. uberis* could be detected in this limited extent.

### 3.4 Time-kill assay of potential antimicrobials

#### 3.4.1 *Klebsiella* species

In Table 7, results of the time-kill assay of NAC, NCT, and MGO for *K. oxytoca* and *K. pneumoniae* are listed.

**Table 7:** Results of time-kill assay of three potential antimicrobial substances for one *Klebsiella oxytoca* and one *Klebsiella pneumoniae* isolate and reduction of bacterial concentration compared to positive control.

Substance		*K. oxytoca*		*K. pneumoniae*	
	Time	Concentration	Time-dependant reduction	Concentration	Time-dependant reduction
NAC	2	6.5	98.98	6.5	97.56
	5	6.5	99.99	6.5	99.87
NCT	2	47	3.98	47	0
	5	47	99.06	47	0
	10	47	100	47	99.99
MGO	2	28	87.70	30	0
	5	28	99.95	30	73.82

Time in minutes, concentrations in mg/mL and reduction of bacterial concentration in % compared to positive control; *n* = 1 for every combination of substance and isolate; NAC, N-acetylcysteine; NCT, N-chlorotaurine; MGO, methylglyoxal.

Differences in time period and reduction can be found between the substances: while NAC inhibits bacterial growth already at 2 min around 98 to 99%, NCT and MGO need five (for *K. oxytoca*) to 10 min (*for K. pneumoniae*) to display their full antimicrobial activity.

Based on this experiment, MBC of NCT for *K. pneumoniae* is at 47 mg/mL and for *K. oxytoca* between 45 and 47 mg/mL and a MBC of MGO for *K. oxytoca* around 28 mg/mL can be found.

#### 3.4.2 Streptococcus uberis

Results of the time-kill assay of NAC, NCT, and MGO for *S. uberis* are shown in Table 8.

**Table 8:** Results of time-kill assay of three potential antimicrobial substances for one *S. uberis* isolate and reduction of bacterial concentration compared to positive control.

Substance		*S. uberis*			
	Time	Concentration	Time-dependant reduction	Concentration	Time-dependant reduction
NAC	2	3.75	0	4	0
	5	3.75	72.67	4	82.98
	10	-	-	4	99.73
NCT	2	40	45.1	45	0
	5	40	85.98	45	89.92
	10	-	-	45	99.98
MGO	2	23	0	25	0
	5	23	0	25	60.75
	10	23	99.99	-	-

Time in minutes, concentrations in mg/mL and reduction of bacterial concentration in % compared to positive control; −, combination not examined; *n* = 1 for every combination of substance and isolate; NAC, N-acetylcysteine; NCT, N-chlorotaurine; MGO, methylglyoxal.

All three substances enrolled their complete antimicrobial abilities not until ten minutes of incubation while the increase of the reduction rate is higher between minutes 2 and 5 and flattens from minutes 5 to 10. A higher concentration led to a higher reduction rate at the same time points.

## 4 Discussion

The primary goal of this study was to evaluate the *in vitro* efficacy of four potential non-antibiotic antimicrobials — PVP-iodine, NAC, NCT, and MGO — against *Klebsiella* spp. and *S. uberis* isolates from bovine mastitis. To contextualize these findings, we first characterized the antimicrobial susceptibility of the isolates to a panel of conventional antibiotics using the broth microdilution method, the gold standard for such assessments (27). This initial step was not intended to provide a comprehensive epidemiological overview but rather to position our isolates within the broader landscape of antimicrobial resistance and establish a baseline for comparison with the novel substances. To our knowledge, this is the first study that examines MICs of the four potential non-antibiotic microbials PVP-iodine, NAC, NCT, and MGO against clinical isolates of bovine mastitis pathogens *Klebsiella* spp. and *S. uberis* and compares them to the susceptibility of the isolates to conventional antibiotics.

### 4.1 Antimicrobial susceptibility

The generated MICs of antibiotic agents were compared to data from studies determining antimicrobial susceptibility with broth microdilutions. We excluded data from studies with disc diffusion, as results of both methods agree only by 80% for mastitis pathogens, partly due to non-specific breakpoints (30). Generally, only a few studies examined antimicrobial susceptibility of *Klebsiella* spp. isolated from bovine mastitis. Therefore, we found no MICs from penicillins, macrolides, and lincosamides, which may not be surprising considering intrinsic resistances. Additionally, due to differing concentration ranges examined, not all MICs were fully comparable. Results of studies from Germany, Europe, and China were regarded. Investigated *Klebsiella* spp. isolates showed MICs below examined concentrations of the following classes of antibiotic agents and were therefore found to be susceptible to aminopenicillins combined with beta-lactamase inhibitors, cephalosporins (partly combined with aminoglycosides) and fluoroquinolones (Table 1; Supplementary Table 3). In contrast, MICs of benzylpenicillins, aminopenicillins, isoxazolyl-penicillins, macrolides, and lincosamides were higher than investigated concentrations of antibiotic agents (Table 1; Supplementary Table 3) and *Klebsiella* spp. isolates were considered not susceptible. One *K. oxytoca* isolate was only susceptible to amoxicillin/clavulanic acid, cefoxitin, cefquinome, and marbofloxacin (Table 1; Supplementary Table 3). Intrinsic resistance of *Klebsiella* spp. to ampicillin and erythromycin (31) was confirmed by our findings. Regarding susceptibility to single antibiotic agents, MIC for amoxicillin/ clavulanic acid was ≤8/4 μg/mL. In national and international studies, MIC_50_ was between 2 μg/mL (32–34) and 4 μg/mL (8, 20), whereas MIC_90_ varied from 4 μg/mL (8, 33) to 64 μg/mL (34), which is a bimodal distribution, pointing at evolving resistances. In accordance with the MIC distribution, Song et al. found 17% amoxicillin/ clavulanic acid resistant *Klebsiella* spp. isolates in China (34), while resistance rates in Europe decreased from 4.6% (2009) to 1.4% (2016), just as MIC_90_ dropped from 8 μg/mL to 4 μg/mL over the same time (32, 33). The combination of aminoglycoside and a first-generation cephalosporin, kanamycin/cefalexin, had a MIC from ≤2/0.2 μg/mL, similar to MIC_50_ found in Europe (32, 33). A higher MIC_90_ with 4 μg/mL (32, 33) and resistance rates of *Klebsiella* spp. from around 6% for cephapirin to 9% for cefazolin, two first-generation cephalosporins, were reported in Europe too (32, 33). A third-generation cephalosporin, ceftiofur, had a MIC of ≤2 μg/mL, similar to a monomodal MIC_50/90_ of 0.5 μg/mL (32, 33) in Europe. In contrast, MIC_50_ of 0.5 μg/mL and MIC_90_ of 16 μg/mL (34) in China showed a bimodal distribution, implicating emerging resistances. For cefquinome, a fourth-generation cephalosporin, and the fluoroquinolone marbofloxacin, MICs were very low in Germany (8, 20) and Europe (32, 33) with MIC_50/90_ between 0.03 and 0.5 μg/mL, similar to our findings with ≤2 μg/mL and ≤1 μg/mL, respectively. Overall, our MICs of *Klebsiella* spp. isolates where close to or even lower than MIC_50/90_ found in the presented studies.

The predominant gram-positive pathogen, *S. uberis,* showed median MICs below tested concentrations of penicillins, cephalosporins, macrolides, lincosamides, and fluoroquinolones (Table 2; Supplementary Table 4) and was therewith susceptible to all examined antibiotic agents.

The susceptibility to single antibiotic agents, for example penicillin G, was with a MIC of ≤0.125 μg/mL identical with MIC_50_ found in studies from Germany (20), Switzerland (35), Europe (32, 33, 36) and the United States (37). MIC_90_ was higher and differed from 0.25 μg/mL (20, 32, 33, 36) to 4 μg/mL found by Watts (37) in the United States. Interestingly, MIC_90_ decreased in Germany from 2 μg/mL (2002) to 0.5 μg/mL (2021) (21), while it remained constant in Europe (32, 33, 36). These findings hint at a bimodal distribution and go along with reported resistance rates of *S. uberis* against penicillin G from 6% worldwide, calculated in a review by Miotti et al. (38). For oxacillin, MIC was ≤0.25 μg/mL and thereby lower than recorded MIC_50/90_ in Switzerland (1 μg/mL) (35) and northern Germany (2 μg/mL) (20). The BVL found a similar MIC_50_, but a higher MIC_90_ (2 μg/mL) (21). Similarly, MIC for cefoperazone with ≤0.5 μg/mL was lower than MIC_50/90_ found in Switzerland (2 μg/mL) (35) and northern Germany with MIC_50_ 1 μg/mL and MIC_90_ 4 μg/mL (20). Concerning macrolides, MICs for erythromycin showed a bimodal distribution in all studies: our MIC (≤0.25 μg/mL) was similar to MIC_50_ found in Germany (39), Europe (32, 33, 36), United States (37), and Canada (40). MIC_90_ with 64 μg/mL was profoundly higher in most studies (32, 33, 36, 37, 39) except for Canada with 2 μg/mL (40). Again, the BVL found a decrease in MIC_90_ from 32 μg/mL (2002) to 0.06 μg/mL (2021) in Germany (21). As expected through the distinct bimodal distribution, resistance rates from *S. uberis* to erythromycin from around 20% in Europe (32, 33, 36) and 15% worldwide (38) were reported. With regard to lincosamides, distribution of MICs for pirlimycin was bimodal in all studies too. In Germany (39), Switzerland (35), Europe (32, 33, 36), United States (37), and Canada (40), MIC_50_ was close to our MIC (≤2 μg/mL). On the contrary, MIC_90_ varied from >2 μg/mL (40) to 32 μg/mL (37). Pirlimycin resistance was recorded for 16% of *S. uberis* strains in Europe (33) and 6% in Germany (21). MIC of Marbofloxacin with ≤1 μg/mL was similar to MIC_50/90_ found by (35) in Switzerland, while it was lower than MIC_90_ found by BVL from 2002 to 2021 in Germany (2 μg/mL). In Europe, MIC_50_ of 1 μg/mL and MIC_90_ of 2 μg/mL were stable over 14 years (32, 33, 36).

Comparing MICs of *S. uberis* isolates to MIC_50_ and MIC_90_ from national and international studies, only little deviations appear. In general, MICs of *S. uberis* isolates were identical with or even lower than MIC_50/90_ reported in the selected studies.

### 4.2 Potential antimicrobial substances

The first of our four examined substances, PVP iodine, is a commonly used antiseptic in all medical fields. Its chemical structure, a complex formation with molecular iodine (I_2_), enhances antimicrobial activity due to simplified membrane transport. The cytotoxicity seems to depend on concentration and tissue type, while diluted formulations have a better biocompatibility (23). In surgery, conventional concentrations of 5–10% PVP iodine are used for aseptic preparation of operation fields. More frequent developments use more dilute concentrations, for example 0.25% PVP iodine applied every 30 s during cataract surgery (41). The paradox increase of efficacy of diluted PVP iodine concentrations can be explained through kinetics: an equilibrium between complex bound and free iodine develops, while more iodine is released from the complex at low concentrations (23). Therapeutic use in veterinary medicine includes bovine endometritis, where intrauterine infusions of 2% PVP iodine solution were effective (42). Although causing transient uterine inflammation, Yoshida et al. found a promoted regeneration of epithelial cells and improved fertility (43). Regarding use of PVP iodine in context with bovine mastitis, only few studies were conducted. Most of them focused on efficacy of dipping agents: Skowron et al. examined bacterial load on teat skin fragments incubated in five common mastitis pathogens, amongst them *K. pneumoniae,* and afterwards treated with dipping agents. They found reduction rates of 99.35 to 99.96% of bacterial load through PVP iodine (44). Furthermore, examinations of algaecide concentrations of different dipping agents, like PVP iodine, against *Prototheca* isolated from bovine milk were carried out (45). A less therapeutic but more radical attempt was permanent cessation of lactation of quarters infected with *Staphylococcus aureus* (*S. aureus*) with 5% PVP iodine solution, which lead to eradication of the pathogen but also loss of the functionality of the treated quarter (46). Optimal bactericidal concentrations from 0.08–0.9% PVP iodine were found in a review by Sauerbrei (47), which goes along with our MICs of 5 mg/mL for *Klebsiella* spp. (Table 3) and 4 mg/mL for *S. uberis* (Table 4).

The acetylated derivate of the amino acid L-cysteine, NAC, has cytoprotective, anti-inflammatory and antimicrobial effects (24). It is a precursor of glutathione, an important antioxidant, and leads to decreased concentrations of proinflammatory tumor necrosis factor alpha (TNF-*α*) and interleukins (IL-6 and IL-1ß). Reduction of disulfide bonds, that is, in the mucus, and inhibition of biofilm formation are further properties (24). In human medicine, it is used as mucolytic agent and antidote to acetaminophen intoxication (48). Studies in this field found a reduction of bacterial load in existing biofilms (combined with ciprofloxacin) and cell invasion of uropathogens in presence of NAC (49). Additionally, healing of wounds infected with *S. aureus* was improved due to treatment with NAC (50). Investigations related to veterinary medicine showed abilities in decreasing apoptosis and autophagy in RAW cells challenged with mycolic acid (51). With focus on therapeutic use in cattle, examinations regarding bovine endometritis proved higher clinical cure rates in groups treated with NAC (77.2%) compared to the non-NAC group (43.3%). Furthermore, the NAC group showed enhanced gestation rates (66.7%) in contrast to the non-NAC group (54.6%) (52). Against bovine mastitis pathogens, it served as an antimicrobial adjuvant, as 10 mM NAC led to lower MICs of penicillin and ampicillin but higher MICs of erythromycin and ciprofloxacin for all examined bacterial strains (53). In a follow up study, Yang et al. (54) found decreased MICs of ß-lactam antibiotics against MRSA from bovine mastitis cases.

We focused on the antimicrobial activity of NAC alone and received MICs of 5 mg/mL for *Klebsiella* spp. (Table 3) and 1.75 mg/mL for *S. uberis* (Table 4). Similarly, Brinkmann (55) obtained MICs of 3.5 mg/mL against *E. coli* isolates from bovine mastitis. In experiments with multi-resistant *Staphylococcus pseudintermedius (S. pseudintermedius)* isolated from dogs, Bohn et al. (56) found MICs of 2.5 mg/mL. Keratitis associated *S. pseudintermedius* and *Pseudomonas aeruginosa* (*P. aeruginosa*) from dogs had a MIC of 3.12 mg/mL, compared to 1.56 mg/mL for *Streptococcus canis* (57). MBCs ranged within the same level, around 3.75 mg/mL for *S. uberis* and 6.5 mg/mL for *Klebsiella* spp. Interestingly, bactericidal activity of NAC against *Klebsiella* spp. seems to evolve earlier than for *S. uberis,* as preliminary results of time-kill assays showed an effect after 2 min against *Klebsiella* spp. (Table 7) compared to 10 min against *S. uberis* (Table 8). Regarding its pharmacokinetic properties, the carboxyl group with a negative charge at physiological pH, hinders passive transport across cell membranes (58). *In vivo,* a high first pass effect occurs when administered orally (59). Oral application goes along with less side effects than intravenous application, correlating with higher plasma levels of NAC (60). Consequently, NAC is a promising antimicrobial with abilities in a wide range of indications, for both human and veterinary medicine and against diverse bacterial strains.

The second amino acid derivate, NCT, originates from taurine and is synthesized endogenously by activated human granulocytes and monocytes. Belonging to long-lived oxidants (N-chloro-derivates of amino acids), it has anti-inflammatory properties and controls inflammatory response. Efficacy against bacteria, fungi, viruses, and parasites has been proved. The synthetic sodium salt enables storage and targeted therapeutic use. A 1% aqueous solution is eligible for application on eyes, skin ulcerations, the outer ear canal, nasal sinuses, oral cavity or urinary bladder. Therapeutic use in human medicine includes external otitis, keratoconjunctivitis and purulent skin ulcerations. Additionally, clinical studies showed high tissue tolerability (25).

Only one study examined applicability in veterinary medicine for bovine mastitis. NCT solution in distilled water in three concentrations (0.1, 1, 2%) was administered intramammary in healthy cows over 5 days. Parallel, the control group received 0.9% NaCl solution. Local and systemic effects were monitored. No increase of taurine levels in blood and urine was observed, which disproved a systemic distribution of intramammary applied NCT. Minor local effects like slight indurations of quarters and a marginally reduced milk yield were observed in both, the NCT and the control group. Leucocyte counts in milk increased 2 days after application in the NCT group and on day 3 in the saline group as well, with no statistical difference between the groups. 5 days after end of treatment, leucocyte counts were back to the baseline in both groups. Examinations regarding residues in milk found no more oxidation capacity, caused by NCT, in milk maximally 5 h after application. In addition to the *in vivo* experiment, Huber et al. (61) examined the *in vitro* reduction of *E. coli* and *S. aureus* in cow milk. After 10 respective 20 min, a 1% (= 10 mg/mL) NCT solution reduced bacterial concentration, measured in colony forming units per milliliter, by four to five log_10_ steps. This outcome was not observed for 0.1 and 0.01% NCT solutions. The authors concluded that except for slight local side effects, NCT could be a safe and effective antimicrobial agent in bovine mastitis therapy (61).

Interestingly, our MICs of NCT for *Klebsiella* spp. were 1.5 mg/mL (Table 3) and 1 mg/mL for *S. uberis* (Table 4) and therefore in the same concentration range as the ineffective dose from Huber et al. (61). This observation may be explained by the effect described by Gottardi and Nagl (25), that due to substantial amounts of reducing substrates in culture media, in this case milk, examinations display too high NCT concentrations for the desired effect. Possibly, cow milk contains more reducing substrates than CAMBH, which may lead to the difference in effective NCT concentrations. Regarding MBCs of NCT, they appear massively higher than detected MICs (1.5 mg/mL compared to 40–47 mg/mL).

The last tested substance, MGO, is one of the antimicrobial components in Manuka honey (26), originating from nectar of *Leptospermum scoparium,* a plant native to New Zealand and Australia (62). Manuka honey’s present therapeutic use is mainly in wound gels (63). Synergistic effects with antibiotics, like rifampicin (64), linezolid against *S. aureus* (65), and piperacillin, amikacin, and carbenicillin against *P. aeruginosa* (66), were reported for the isolated component MGO.

Furthermore, treatment with MGO alone lead to inhibition of growth of *P. aeruginosa* (67), *S. aureus* and *E. coli* (68) and inhibition of biofilm formation of *P. aeruginosa* and methicillin-resistant *S. aureus* (69). Its antimicrobial effects result from binding of proteins, lipids, and nucleic acids, leading to formation of advanced glycation end products and loss of functionality (70), for example in bacterial fimbriae and flagellar proteins, interfering with bacterial adherence and motility (71).

In a study with MGO as additive to bone cement against multi-resistant *S. pseudintermedius* isolated from dogs, 25 mg MGO per platelet decreased bacterial colonization. Additionally, reduced infection of HOS cells was achieved by 0.05 mg/mL MGO. Limited biocompatibility with an IC_50_ of 0.17 mg/mL MGO for cell viability was a challenge for therapeutic use, as it was very close to the MIC (0.15 mg/mL) (72). This result is similar to our MIC for *S. uberis* (0.1375 mg/mL) (Table 4), but noticeably lower than MIC for *Klebsiella* spp. (0.4 mg/mL) (Table 3). Furthermore, MBCs for *S. uberis* (23 mg/mL) are below them for *Klebsiella* spp. (28 mg/mL). The differences may be due to distinct composition of cell walls of gram-positive and gram-negative bacteria. Comparing MICs and MBCs, MBCs are immensely higher. This may point at a mainly bacteriostatic mechanism of MGO in inhibition of bacterial growth, although bactericidal properties could be observed too.

Limitations of this study are for example within the small sample size and regionally restricted sample collection. Furthermore, the lack of examinations on biocompatibility and in more complex settings, for example other media like milk, constrain the generalization of our results.

### 4.3 Outlook and conclusion

Still, this study successfully demonstrated the *in vitro* antimicrobial activity of PVP-iodine, NAC, NCT, and MGO against key bovine mastitis pathogens, *Klebsiella* spp. and *S. uberis*. Crucially, these substances were effective even against bacterial isolates that exhibited resistance to conventional antibiotics. These findings, amended with results from continuative examinations, position them as auspicious candidates for alternative mastitis therapies.

The path forward requires a multi-step approach. The immediate next step is to rigorously evaluate the biocompatibility of these substances using primary bovine mammary epithelial cells to determine their cytotoxic profiles. Subsequently, *in vitro* and *ex vivo* infection models, such as precision-cut bovine udder slices (PCBUS), will be essential to assess their efficacy in a more physiologically relevant environment that mimics tissue conditions. These advanced models will help determine whether a safe and effective therapeutic window can be achieved *in vivo*, paving the way for potential clinical trials and the development of novel, non-antibiotic treatments for bovine mastitis.

## Data Availability

The original contributions presented in the study are included in the article/Supplementary material, further inquiries can be directed to the corresponding author.
